# Spina Bifida

**Published:** 1854-12

**Authors:** 


*Spina Bifida, a new plan of treatment by* Mr. Paget.

From the peculiar nature of this affection, owing to the almost invariable implication of the spinal cord, or its nervous branches, as have been satisfactorily shown by Cruveilheir, Stafford, Prescott, Hewitt, and other writers, we cannot expect that any surgical operation will ever prove successful, unless in certain cases in which the nerves of the cord have no connection with the sac of the tumor, or where very few branches are in connection with it, or where the tumor may be so small that its opening of communication with the spinal canal is in accordance with the size of the tumor itself. Of 20 cases examined by Mr. Hewett in various collections, only one was free from contact with the spinal nerves, and Cruveilheir again believes, from his dissections, that the connection between the tumor and the nerves is constant. This testimony, of course, invalidates very much against operative procedure. We shall find, however, on reference to the medical journals, that cases are occasionally cured, and many modes of treatment have been adopted with or without success; I may mention, for instance, pressure by means of a hollow pad or truss, puncturing the sac, ligature around the tumor, injections of iodine, removal, or any of these combined. Dr. Brainard, of Chicago, U. S., cured 1 out of 4 cases by the iodine injections, and Velpean and Chassaignac have treated cases with great success by the same treatment, on the testimony of the Gazette des Hopitaux. Puncture and pressure in certain cases offer the best, and, in fact, the only means of cure.

The following case presents some points of interest in being operated upon in a totally different manner from any case hitherto recorded, by Mr. Paget, on the 15th July, at Bartholomew's Hospital. The patient was a stout, healthy child, 3 months old, with a tumor the size of a large fœtal head, situated over the lower dorsal and upper lumbar vertebræ. A sub-cutaneous ligature was passed around the base of the tumor, with its two ends emerging from the superior margin of its base; these were fastened to two India rubber straps, which crossed the shoulders, and which were kept in position by a wide and long band of adhesive plaster passed around the chest. It appears that pressure upon the tumor does not in any way affect the cerebral functions of the child, and Mr. Paget concludes from this and other reasons, that the opening of communication between the cyst and spinal cord most probably is very small, and therefore favorable to the operation. His object in applying the ligature under the skin, and fasteniug the ends to the India rubber straps, is to permit of the thread cutting its way out, and thus isolating the cyst, a result likely to happen in about 14 days. Should this succeed, he will be prepared to perform another operation for the removal of the cyst. Under any circumstances, this disease is almost always fatal, and the present operation is merely an experiment which suggested itself to his mind, and he believes it may prove successful. Should it not, we are still at liberty, he says, to try something else. The child was not put under the influence of chloroform, which I candidly think was a great omission.

Since the foregoing was written, the irritation and pain from the ligature became so great as to cause the child much suffering, which ended in death four days after the operation.

